# Medial Septum Modulates Consciousness and Psychosis-Related Behaviors Through Hippocampal Gamma Activity

**DOI:** 10.3389/fncir.2022.895000

**Published:** 2022-07-07

**Authors:** L. Stan Leung, Jingyi Ma

**Affiliations:** Department of Physiology and Pharmacology, University of Western Ontario, London, ON, Canada

**Keywords:** gamma waves, ketamine, general anesthesia, hippocampal seizure, schizophrenia

## Abstract

Abnormally high-amplitude hippocampal gamma activity (30–100 Hz) in behaving animals is seen after a hippocampal seizure, following injection of phencyclidine (PCP) or ketamine, and transiently in a delirium stage during induction of general anesthesia. High-amplitude hippocampal gamma activity in behaving rats is associated with hyperactive behavior and impairment in sensorimotor gating and sensory gating. The medial septum is necessary for the high-amplitude gamma activity and abnormal behaviors observed following a hippocampal seizure or injection of PCP/ketamine. Glutamatergic projection of the hippocampus to the nucleus accumbens (NAC) and dopaminergic transmission in NAC is necessary for abnormal behaviors. Large hippocampal gamma waves are suggested to contribute to seizure-induced automatism following temporal lobe seizures, and the schizophrenia-like symptoms induced by PCP/ketamine. Low-amplitude gamma activity is found during general anesthesia, associated with loss of consciousness in humans and loss of righting reflex in animals. Local inactivation or lesion of the medial septum, NAC, and brain areas connected to the septohippocampal-NAC system attenuates the increase in hippocampal gamma and associated behavioral disruptions induced by hippocampal seizure or PCP/ketamine. Inactivation or lesion of the septohippocampal-NAC system decreases the dose of anesthetic necessary for gamma decrease and loss of consciousness in animals. Thus, it is proposed that the septohippocampal-NAC system serves to control consciousness and the behavioral hyperactivity and neural dysfunctions during psychosis.

## Introduction

### Consciousness and Impaired Awareness in Seizures and Schizophrenia

The medial septum modulates hippocampal local field potentials (LFPs) including the 30–100 Hz gamma activity. Low- and high-amplitude hippocampal gamma activity are associated respectively with low and high activation of the hippocampus and behavior. The lowest gamma activity is found during deep surgical anesthesia, with loss of consciousness (Leung et al., 2014). The middle range of gamma amplitudes is associated with normal behavioral states ranging from slow-wave sleep, awake-immobility, and from small movements to increasingly vigorous voluntary movements (Leung, 1998). The largest hippocampal gamma in animals has been recorded after a hippocampal seizure or after acute administration of ketamine/ phencyclidine (PCP). Abnormally large gamma is associated with behavioral hyperactivity and psychosis-related behaviors in animals (Ma et al., 2004).

Level of consciousness may be indicated by the amplitude of hippocampal gamma waves. Consciousness, defined as awareness of self and environment in humans, is lost during general anesthesia (Franks, 2008), although unresponsiveness without losing awareness has been suggested. The surrogate for loss of consciousness in animals is loss of righting reflex (LORR), since the dose of general anesthetic that induces LORR is highly correlated with the dose that induces loss of consciousness in humans (Franks, 2008). Consciousness is said to be impaired after a complex partial seizure, now called a focal seizure with impaired awareness by the International League Against Epilepsy (Falco-Walter et al., 2018). Given that consciousness can only be inferred from the behavior and physiology of an animal, it may be more appropriate to describe the neurobiological processes that are altered after seizures, such as perception, memory, voluntary movements, and affect (Gloor, 1986).

Schizophrenia is a mental disorder characterized by positive symptoms of hallucination, delusion, and thought disorder. Schizophrenia has been described as excessive self-awareness (Frith, 1979), or as a failure to integrate stored memories with ongoing motor programs (Gray et al., 1991). Other than behavioral hyperactivity and cognitive deficits, animal models of schizophrenia have been evaluated with paradigms of sensory gating and sensorimotor gating. Gating deficits are considered to be neural processing deficits in a schizophrenia condition in humans and animals (Adler et al., 1985; Swerdlow et al., 2008). Acute schizophrenia can be induced by ketamine, an N-methyl-D-aspartate (NMDA) receptor antagonist, in humans and animals (Moghaddam and Krystal, 2012).

### Septohippocampal System in Brain Arousal

**Figure 1:**
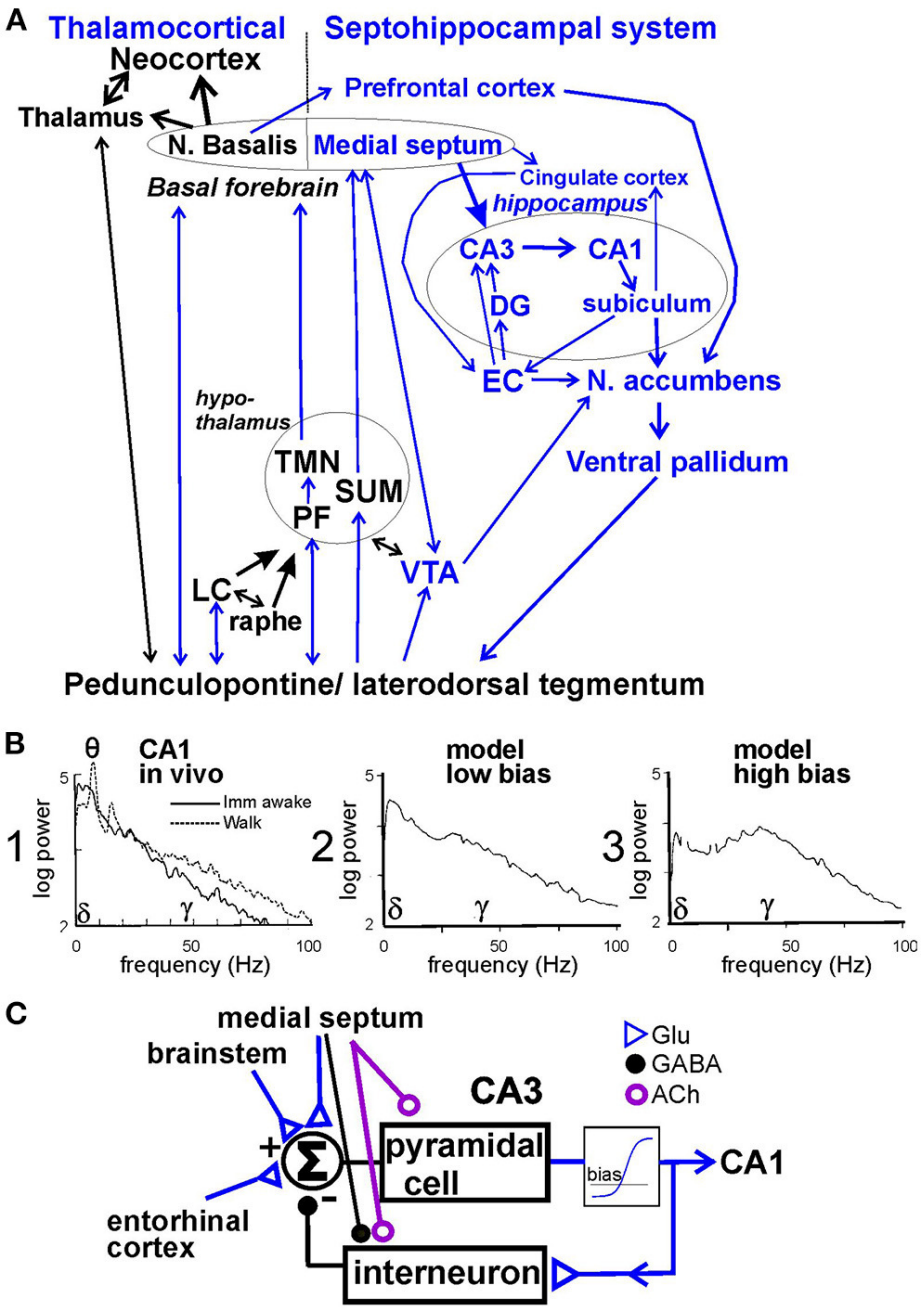
**(A)** Schematic neural circuit for arousal and psychotic behaviors in septohippocampal and thalamocortical systems. Peduncolopontine tegmentum and lateral dorsal tegmentum project to monoaminergic nuclei [locus ceruleus (LC), raphe, and ventral tegmental area (VTA)] and hypothalamic areas [perifornical area (PF), tuberomammillary nucleus (TMN), and supramammillary area (SUM)] which also project to the basal forebrain (medial septum and n. basalis) and thalamus. The limbic cortices of hippocampus, entorhinal cortex (EC) receive afferents from the medial septum; cingulate and prefrontal cortices receive afferents from both the medial septum and n. basalis. Limbic cortices project to the nucleus accumbens (NAC) and then ventral pallidum, and back to the brainstem. NAC and ventral pallidum mediate behaviors. **(B)** 1. Power spectrum of hippocampal local field potentials recorded in CA1 stratum radiatum during awake immobility (imm) and walking in the rat; power was measured in (mV)^2^/Hz, and theta peak power corresponds to a theta amplitude of ~ 1 mV peak-to-peak. 2. Hippocampal residue spectrum (without theta peaks) is generated by model with low bias; and 3. with high bias. **(C)** Recurrent feedback model of pyramidal cells exciting inhibitory interneurons through a non-linear element, with bias level determined by background activity, and feedback gain modulated by the medial septum, adapted from Leung (1982). Glu, glutamatergic; GABA, gamma aminobutyric acid, ACh, acetylcholine.

The neural circuits for brain arousal have been reviewed (Saper et al., 2010). We have suggested that the arousal circuit consists of two components—thalamocortical and septohippocampal systems (Leung et al., 2014; Figure 1A). Both systems are activated by wake-active areas in the brainstem, among them the peduculopontine tegmentum (PPT), laterodorsal tegmentum (LDT), monoaminergic nuclei such as locus ceruleus (containing neurons with norepinephrine), raphe nucleus (serotonin), ventral tegmental area (dopamine), and hypothalamic nuclei such as tuberomammillary nucleus (histamine), perifornical nuclei (orexin) and supramammillary nucleus. The basal forebrain receives afferents from diverse wake-active areas, and provides behavioral activation to the cerebral cortex. Within the basal forebrain, the medial septum/ diagonal band of Broca complex projects to the hippocampus, entorhinal and cingulate cortices, while the basal nucleus of Maynert projects to the neocortical mantle.

The connection of the hippocampus to the nucleus accumbens (NAC) and ventral pallidum has been suggested to translate motivation to movements (Mogenson et al., 1993). Septohippocampal-NAC circuit is implicated in mediating schizophrenia-like behaviors in animals (Gray et al., 1991). In this review, we suggest that the medial septum, hippocampus and NAC are involved in linking abnormally high hippocampal gamma waves with psychosis-related behaviors, following administration of a hippocampal seizure or low-dose ketamine.

### Hippocampal Theta and Gamma as Measures of Activation and Memory

Arousal and behavior are associated with the hippocampal LFPs. Hippocampal theta rhythm, of range 3−12 Hz in rodents, correlates with the moment-to-moment behavior of animals (Vanderwolf, 1969). The level of arousal is revealed by the frequency of theta rhythm, which increases with intensity of brainstem stimulation (Vertes, 1981; Oddie et al., 1996), together with an increase in firing of many wake-active brain areas from quiet to active waking (Leung et al., 2014; Jones, 2020). A tight correlation of septal modulation of behaviors is demonstrated by the following experiment. Oddie et al. (1996) showed that following procaine infusion into the medial septum of rats, hippocampal theta rhythm and wheel-running induced by stimulation of the posterior hypothalamus disappeared at the same time, and then reappeared together as the effects of procaine dissipated.

Hippocampal gamma waves were not recorded by Oddie et al. (1996), but gamma is expected to be correlated with the occurrence of theta and movements (Leung et al., 1982; Leung, 1998). Based mainly on recordings from different layers, hippocampal gamma has been classified as slow (30−60 Hz), high or intermediate (60−100 Hz) and fast (~100–150 Hz) ranges (Scheffer-Teixeira et al., 2012; Fernandez-Ruiz et al., 2017). The oscillatory drive of slow gamma apparently originates from CA3 (Bragin et al., 1995; Csicsvari et al., 2003; Fernandez-Ruiz et al., 2017), while that of intermediate or fast gamma is suggested to come from the entorhinal cortex (EC; Colgin et al., 2009; Scheffer-Teixeira et al., 2012). Ablation of the EC increased the CA3-generated gamma waves of 30–60 Hz at the CA1 proximal apical dendrites by several fold, which was interpreted as a release of dentate suppression of CA3-CA1 gamma after EC ablation (Bragin et al., 1995).

Hippocampal activation in behaving rodents, as observed in the LFPs and evoked potentials (Leung et al., 1982; Leung, 1985, 1998; Leung and Chu, 2021), ranks from low activation during slow-wave sleep and awake-immobility, intermediate activation during small (e.g., head) movements and high activation during vigorous voluntary movements (walking, turning, and rearing; Figure 1B1). With the lowest activation, hippocampal LFPs show large irregular activity, or high-amplitude slow waves (<25 Hz) accompanied by low-amplitude gamma (30−100 Hz) activity (Figure 1B1; Leung, 1985, 1998). Small activation decreases irregular slow waves with small or no theta and gamma increase, and high activation increases theta and gamma oscillations (Figure 1B1; Leung, 1985, 1998; Leung and Chu, 2021). In the hippocampus, the residual power spectrum, or power spectrum without theta rhythm, gives a better description of the activation at low levels, since a prominent theta rhythm only accompanies moderate and high activation in rodents.

Gamma oscillations in the hippocampus are suggested to participate in neural processing and communication. One model describes that about 7 gamma cycles within half a theta cycle represent clock cycles in processing sensorimotor data during the formation or retrieval of an episodic memory (Lisman and Buzsáki, 2008; Lisman and Jensen, 2013). Essentially, each event is encoded by an ensemble of neurons, and a time sequence of events is a memory. In other words, each gamma cycle is a computer clock cycle for information processing, working progressively to construct or retrieve a memory of a sequence of sensorimotor events. The coupling of hippocampal CA3 low-gamma (30−60 Hz) amplitude to theta phase is suggested to correlate with performance of a context-dependent discrimination task in rats (Tort et al., 2009).

Our basic model of hippocampal gamma, generated in CA3/CA1, is a recurrent inhibitory circuit consisting of pyramidal cells and parvalbumin-positive inhibitory interneurons, such as basket cells (Freeman, 1975; Leung, 1982; Sohal et al., 2009; Figure 1C). In normal animals, gamma is generated by an intrinsic oscillation in CA3, and the oscillation is projected to CA1 (Csicsvari et al., 2003). CA1 has a similar recurrent inhibitory circuit, but normally less likely to oscillate at a gamma frequency compared to CA3. The intrinsic oscillation depends on a non-linear negative feedback gain, which is the gain around the loop with pyramidal cells exciting interneurons and interneurons inhibiting pyramidal cells (Figure 1C). The feedback gain is effectively zero when pyramidal cells are completely inhibited (firing at zero rate), and it increases quickly depending on an operating bias or the net background activity (Figure 1C). Low and high bias simulate, respectively, low and high activation in CA1 *in vivo* (Figure 1B). Medial septal modulates the hippocampal neurons by cholinergic afferents on pyramidal cells and interneurons, GABAergic afferents on interneurons (Freund and Buzsáki, 1996), and glutamatergic afferents on CA3 pyramidal cells (Robinson et al., 2016). The EC provides glutamatergic inputs to CA3 and CA1, partly through the dentate gyrus (DG; Witter, 2007). For gamma generation in CA3/CA1, extrinsic inputs to CA3/CA1 need not be rhythmic (Leung, 1982).

## Conditions That Increase Hippocampal Gamma Activity

We have studied three main experimental conditions associated with a high-amplitude hippocampal gamma activity and hyperactive, dyscognitive behaviors in animals. The first condition follows the occurrence of an after discharge (AD) or electrical seizure in the hippocampus, induced by electrical or chemical stimulation. The second condition is induced by psychoactive drugs, such as ketamine and PCP that induced psycho-mimetic behaviors at low doses. The third condition occurs transiently after a dose of a general anesthetic, either volatile or non-volatile.

### Post-seizure

#### Electrically Induced Hippocampal Seizures

**Figure 2:**
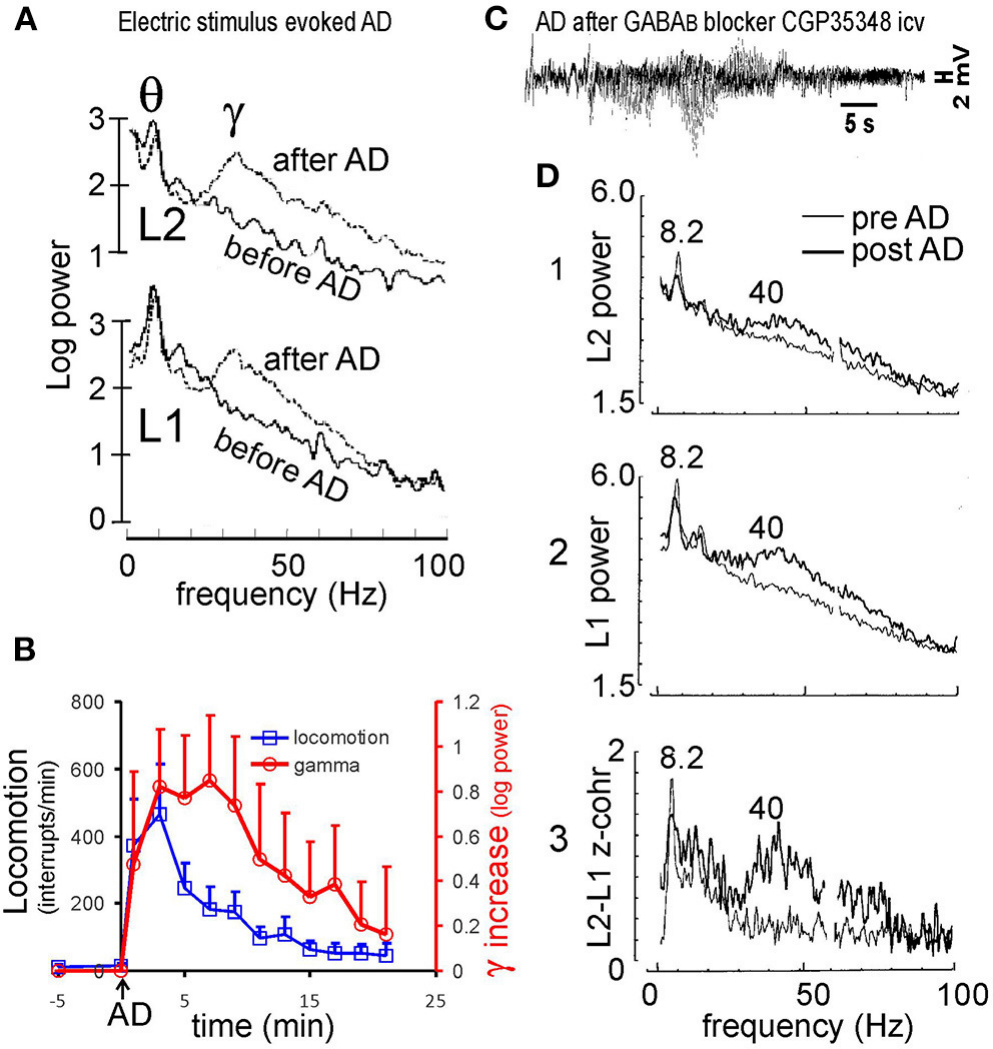
Hippocampal seizure and gamma. **(A)** Electrically induced after discharge (AD) in the hippocampus induced large gamma activity at electrodes across hippocampal CA1 area; L1 electrode placed at stratum radiatum, and L2 at near the alveus. **(B)** Minute by minute horizontal locomotion (infrared beans interrupts/min) and increase in hippocampal gamma (30–60 Hz) from baseline after a hippocampal AD at time 0; mean plus SE (*n* = 4 rats). **(C)** Characteristic pattern of hippocampal AD was observed at 17 min after injection of GABA_B_ receptor antagonist CGP35348 (110 μg intraventricular, icv). **(D)** Power spectra at L1 and L2 electrodes across CA1, and L1-L2 coherence z-transform spectrum, recorded at 13 min after the CGP35348-induced AD (dark traces), overlaid with those during baseline walking (light traces). Power and coherence were high at theta (8.2 Hz) and gamma (peak at ~40 Hz). Power at the theta peak corresponded to ~ 1 mV of theta amplitude at L1 electrode. **(A)** After Leung (1987), **(C,D)** after Leung et al. (2005).

An electrical seizure or AD evoked in hippocampal CA1 was followed a large (2-3-fold) increase in gamma amplitude during normal walking (Leung, 1987; Ma et al., 2004; Figure 2A); the AD must be prolonged (>15 s) in duration. Following a period of postictal depression (when both evoked and spontaneous LFPs were depressed), large gamma activity emerged at 3–20 min after the AD. Postictal gamma power increase in CA1 was found at 30–90 Hz, typically peaking at ~40 Hz (Figure 2A); it manifested as a dipole field, with ~180° phase reversal, in CA1, with little increase of gamma activity in the DG (Leung, 1987). The period of large gamma activity roughly corresponded to a period of hyperactivity (Leung, 1987; Ma et al., 1996; Figure 2B). The group average indicates that the increase in hippocampal gamma persisted longer than that of locomotor activity (Figure 2B). It was also observed that large postictal gamma remained during brief periods of immobility, suggesting a low dependence of postictal gamma on behavior.

Within an hour of an evoked hippocampal AD, rats showed poor spatial working memory in a radial arm maze (Knowlton et al., 1989) and poor acquisition of a hidden platform task in a water maze (Cain et al., 1993). Other than behavioral hyperactivity, sensorimotor gating, as measured by prepulse inhibition of an acoustic startle response (PPI), was impaired during the postictal period (Ma et al., 2004).

The likely cause of high-amplitude postictal gamma activity is a loss of GABA_A_ receptor-mediated inhibition (Kamphuis et al., 1992). In addition, postictal long-term potentiation was found for CA3 to CA1 glutamatergic synapses, in particular at the CA1 basal dendrites (Leung and Shen, 1995). Burchfield et al. (1979) reported an increase in supersensitivity to iontophoretically delivered acetylcholine (ACh) at 40–120 min postictally. Attenuation of the postictal gamma by systemic administration of scopolamine (Leung, 1987) confirmed the participation of muscarinic cholinergic synapses.

Medial septal lesion or inactivation abolished the postictal increase in hippocampal gamma waves (Leung, 1987; Ma and Leung, 1999). Medial septal inactivation was done by infusion of GABA_A_ receptor agonist, muscimol, in the medial septum. Septal inactivation before the hippocampal AD abolished postictal rearing and horizontal locomotion and reduced postictal occurrence of wet dog shake and face wash, without affecting hippocampal AD duration (Ma and Leung, 1999). Septal inactivation immediately after the AD, as compared to control vehicle infusion, also decreased the postictal increase in hippocampal gamma waves and locomotor/ rearing activity (Ma and Leung, 1999). These results suggest that the medial septum continuously modulates gamma waves in the postictal period. However, 192- IgG saporin lesion of the medial septum, which depleted septohippocampal cholinergic neurons (Chang and Gold, 2003), had no significant effect on the postictal increase in gamma or postictal PPI impairment (Ma et al., 2004).

A glutamatergic pathway from the hippocampus to NAC is suggested to induce locomotor activity *via* the ventral pallidum (Groenewegen et al., 1987; Mogenson et al., 1993), including postictal hyperlocomotion (Ma et al., 1996). Bilateral administration of dopamine D2 antagonist (Ma et al., 1996) or metabotropic glutamate receptor antagonist (*RS*)-α-methyl-4-carboxyphenylglycine (MCPG) in the NAC (Ma and Leung, 2002), or bilateral muscimol inactivation of the ventral pallidum (Ma et al., 1996), reduced the increase in behaviors induced by a hippocampal AD. The latter results suggest an action of both glutamate and dopamine in the NAC in the mediation of behavior. MCPG infusion in the NAC did not affect the duration of hippocampal AD or the postictal increase in hippocampal gamma waves, while MCPG infusion in the hippocampus decreased both postictal hippocampal gamma waves and behavioral hyperactivity (Ma and Leung, 2002).

Electrical seizures evoked by stimulation of the basolateral amygdala (Long et al., 2009) or posterior cingulate cortex (Ma and Leung, 2018) did not induce postictal behavioral hyperactivity. However, an electrically induced AD in the mPFC could induce hyperlocomotion, accompanied by gamma waves in the mPFC and NAC (Ma and Leung, 2010). An AD in mPFC was only associated with hyperlocomotion when it also involved the hippocampus, and electrolytic lesion of the hippocampus abolished NAC gamma waves and hyperlocomotion (Ma and Leung, 2010). Apparently, stimulation of glutamatergic afferents from the mPFC to NAC did not induce postictal locomotor hyperactivity without the hippocampus.

#### Chemically Induced Hippocampal Seizures

Chemically induced hippocampal seizures are also known to induce high-amplitude hippocampal gamma waves postictally. The example here shows that a hippocampal AD was induced 17 min after an intraventricular injection of GABA_B_ receptor antagonist CGP35348 in rats (Figure 2C; Leung et al., 2005). The AD had a temporal pattern very similar to that induced by electrical stimulation (Leung et al., 2005), starting with a period of relative electrical silence (~1–2 s) followed by 5–15 s of 2–6 Hz simple spikes (that were negative near the CA1 alveus), and then more complex spikes of mixed polarity (Figure 2C); no generalized convulsions were evoked by the first CGP35348-induced AD. High-amplitude gamma waves started at ~3 min after the AD. The gamma power increase was observed at alveus (L2 electrode) and stratum radiatum (L1 electrode) electrodes in CA1, with ~ 180° phase shift and high coherence across L1 and L2 (Figure 2D). Following the AD, paired-pulse inhibition of the CA1 population spike decreased postictally (Leung et al., 2005), suggesting an inhibition loss. Chemically induced postictal gamma activity was also accompanied by behavioral hyperactivity, but a detailed gamma-behavior relation had not been studied.

#### Postictal Gamma Generation and Medial Septal Effects

Several factors increase the recurrent inhibitory feedback gain in CA3 (Figure 1C) after a hippocampal AD. These include increase in background glutamatergic and cholinergic activity, with underlying mechanisms of cholinergic supersensitivity and glutamatergic long-term potentiation. A high background activity (bias) combined with decreased inhibition of pyramidal cells give a high feedback gain (Figure 1C) and high resonance peak at 30–80 Hz gamma in CA1 (Figure 1B), similar to the postictal gamma (Figures 2A,D). Medial septal inactivation reduces the gamma resonance by removing the septohippocampal muscarinic cholinergic modulation of pyramidal cells, and the septohippocampal inhibition of hippocampal interneurons (Figure 1C). Postictal gamma increase was restricted to CA3 and CA1, and postictal gamma increase in DG was low, suggesting little increase in EC gamma driving. However, persistence of a DG population spike evoked by stimulation of the perforant path suggests that the EC to DG transmission was intact postictally (unpublished data), which may be different from the condition of large CA3 gamma following ablation of the EC (Bragin et al., 1995).

#### Relevance to Focal Seizures of the Temporal Lobe

In patients with temporal lob epilepsy, a single seizure, or more commonly, a cluster of seizures may result in psychiatric symptoms and occasionally in postictal psychosis that lasts for days (Slater and Beard, 1963; Trimble, 1977; Elliott et al., 2009). Complex partial seizures (Gloor, 1986) or focal seizures with impaired awareness are followed by postictal automatism and confusion, similar to the dyscognitive and hyperactive postictal state in animals. Assuming that gamma cycles are related to the retrieval of memory, the abnormally large gamma activity may suggest overactive synaptic activity that may result in encoding or retrieval of altered associations, inappropriate context, and disorganized episodes. Other than amnesia, memory recall problems may contribute to the experience of déjà vu, jamais vu, hallucinations, and delusion, which may occur after complex partial seizures (Gloor, 1986; Elliott et al., 2009).

We are not aware of reports of an increase in 30- 100 Hz gamma activity after a temporal lobe seizure in humans. The typical electrographic activity recorded in an epileptic brain are high-frequency oscillations of 80–500 Hz range (Jacobs et al., 2012). High postictal hippocampal gamma induced by electrical or chemical stimulation is found in otherwise normal animals, while high-frequency oscillations following seizures are recorded in epileptic patients (humans). Postictal depression following a long seizure, and hippocampal cell loss (medial temporal sclerosis) may attenuate postictal gamma increase in epilpetic patients.

### Psychoactive Drugs

#### Non-competitive NMDA Receptor Antagonists

A class of non-competitive NMDA receptor antagonists, including PCP, ketamine and MK-801, induces psychosis symptoms in humans that are indistinguishable from those of schizophrenia (Krystal et al., 1994; Lahti et al., 2001). This class of drugs is pivotal to a paradigm shift that schizophrenia is primarily associated with alteration of glutamatergic NMDA receptors rather than dopaminergic receptors (Javitt and Zukin, 1991; Moghaddam and Krystal, 2012). PCP, ketamine or MK-801 also induces psychosis-related behaviors in rodents, characterized by locomotor hyperactivity, loss of sensorimotor gating (Mansbach and Geyer, 1989; Javitt and Zukin, 1991), and loss of sensory gating (Miller et al., 1992; Miller and Freedman, 1993).

**Figure 3:**
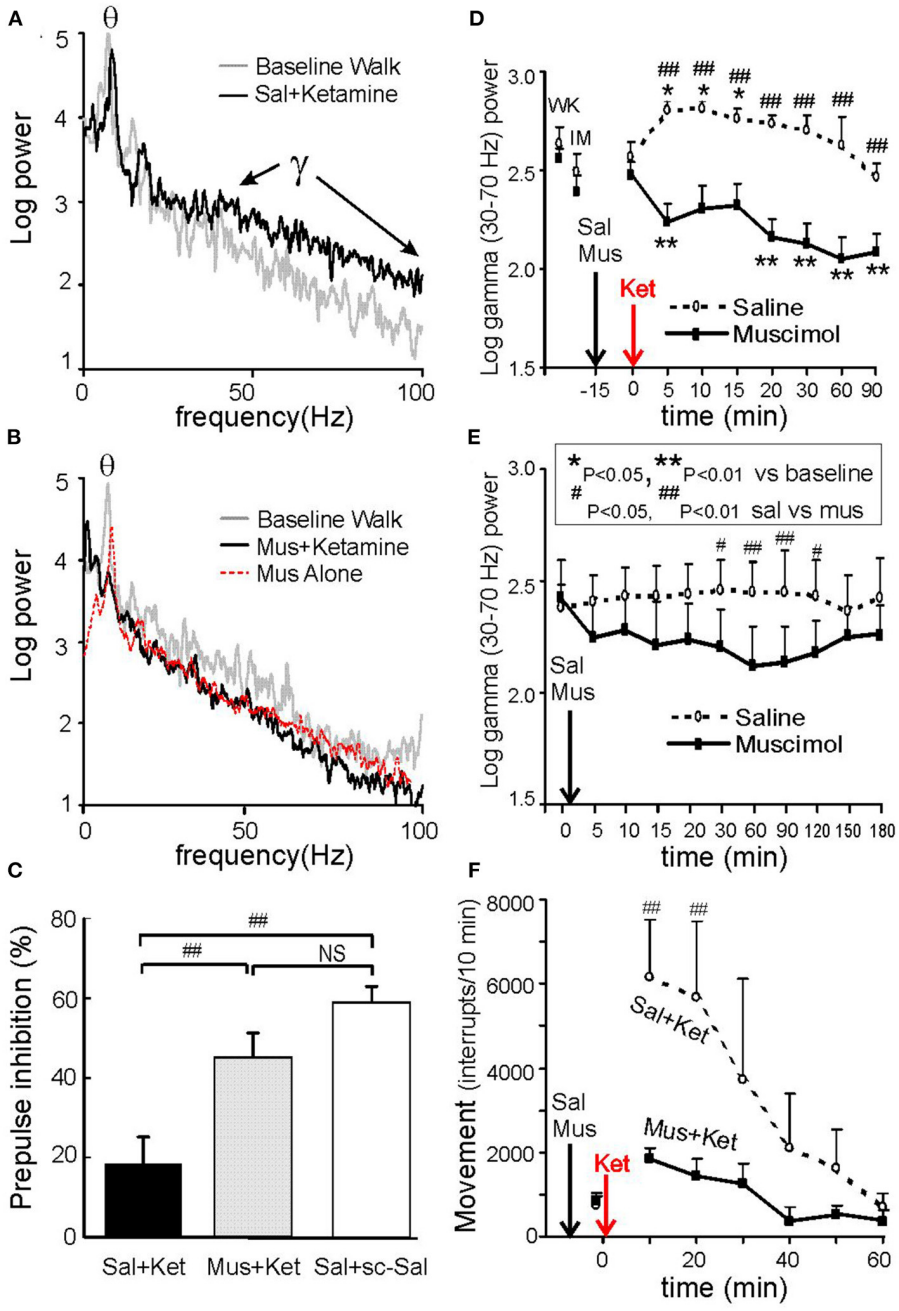
Hippocampal gamma and behaviors induced by 0.6 mg/kg s.c. ketamine and control. **(A)** Power spectra of local field potentials recorded in CA1 stratum radiatum after intraseptal 0.6 μl saline (Sal), compared with baseline walking; note increase of 30–100 Hz gamma after ketamine. **(B)** Same as A except preceded by intraseptal infusion of 0.25 μg/0.6 μl muscimol (Mus) instead of Sal; both theta and gamma were decreased after ketamine; intraseptal Mus only reduced theta peak and 30–100 Hz gamma as compared to baseline. **(C)** Prepulse inhibition (PPI) of the acoustic startle response (10–30 min post-ketamine/ saline s.c.) shows low PPI after intraseptal Sal plus ketamine (Ket) as compared to Sal plus saline s.c.; low PPI induced by Ket was normalized by pretreatment with intraseptal Mus. **(D)** Time course of average 30–70 Hz hippocampal gamma change with time after Ket injection, preceded by either intraseptal Sal or Mus infusion; gamma amplitude of ~0.1 mV = 2 log power units. **(E)** Average gamma (30–70 Hz) change after intraseptal infusion of Sal or Mus only. **(F)** Horizontal movements measured by interrupts of infrared beams show lower movements after septal infusion of muscimol as compared to saline. After Ma and Leung (2007).

Ketamine of 3–6 mg/kg s.c. (Ma and Leung, 2007, 2014; Ma et al., 2009) or 2.5–5 mg/kg s.c. (Hakami et al., 2009) is sufficient to induce schizophrenia-like behaviors in rats, such as locomotor hyperactivity for ~20 min, accompanied by deficits in sensorimotor gating and auditory gating. A comparable ketamine dose in humans is 0.3 mg/kg i.v., which induces schizophrenia symptoms for ~20 min (Lahti et al., 2001). Ketamine (3–6 mg/kg s.c.) induced gamma power increase at 30–100 Hz in CA1 stratum radiatum of rats (Figure 3A; Ma and Leung, 2014). High doses of ketamine, up to a surgical anesthesia dose, were also found to induce large gamma waves in the hippocampus (Leung et al., 2021) and neocortex (Hakami et al., 2009). Other than the hippocampus (Leung, 1985; Ma and Leung, 2000, 2007; Ehrlichman et al., 2009), ketamine also induces large gamma waves in many brain areas, including the posterior cingulate cortex (Ma and Leung, 2018), mPFC, NAC, thalamus, somatosensory, motor, and visual cortices (Pinault, 2008; Hakami et al., 2009; Kocsis, 2012).

Chrobak et al. (2008) reported that ketamine (2.5–100 mg/kg i.p.) disrupted the encoding and retrieval in a delayed-match-to-place radial water maze task. Encoding was affected by 2.5–25 mg/kg i.p. ketamine, and retrieval was affected by 12.5–25 mg/kg i.p. ketamine (Chrobak et al., 2008). Caixeta et al. (2013) showed that 25 mg/kg i.p. ketamine in rats increased the phase-amplitude coupling of hippocampal CA1 theta with gamma of 60–100 Hz, while 75 mg/kg i.p. ketamine decreased theta-gamma coupling. Low gamma (30–60 Hz) was not strongly correlated with theta phase (Scheffer-Teixeira et al., 2012; Caixeta et al., 2013). However, low-dose (<25 mg/kg i.p.) ketamine that induced schizophrenia-like symptoms (above) and poor cognitive performance (Chrobak et al., 2008) with minimally disrupted movements was not studied. Normal animals were reported to show increased coupling of low (30–60 Hz) gamma to theta in CA3 with higher performance in a context-dependent discrimination task (Tort et al., 2009).

Ketamine (6 mg/kg s.c.)-induced hyperactive behaviors and hippocampal gamma waves were abolished by medial septal infusion of 0.25 μg/0.6 μl muscimol (Ma and Leung, 2007; Figures 3D,F). The latter dose of muscimol alone, as compared to vehicle saline infusion, decreased hippocampal theta and gamma at 30–120 min post-infusion (Figures 3B,E). In different experiments, PPI impairment and hyperlocomotion induced by ketamine were normalized by muscimol compared to saline infusion in the medial septum (Figures 3C,F; Ma and Leung, 2007). The disruption of auditory gating in the hippocampus after ketamine was also normalized after muscimol inactivation of the medial septum (Ma et al., 2009).

Hippocampal theta and gamma increase during ataxic walking following (PCP 5–10 mg/kg i.p.) was blocked by a systemic muscarinic cholinergic antagonist (Vanderwolf and Leung, 1983; Leung, 1985). However, lesion of septohippocampal cholinergic neurons by 192-IgG saporin did not affect the hyperlocomotion and PPI deficits induced by PCP (Ma et al., 2004). Selective lesion of septohippocampal GABAergic neurons by orexin-saporin effectively abolished the hyperactive behaviors and hippocampal gamma increase induced by ketamine or MK-801, without abolishing hippocampal theta (Ma et al., 2012).

Deep brain stimulation (DBS) of the medial septum suppressed the increase of hippocampal gamma waves induced by 3 mg/kg s.c. ketamine (Ma and Leung, 2014), and partially normalized the ketamine-induced hyperlocomotion and PPI impairment. One-s on and 4-s off DBS stimulation at 100-Hz was applied to the medial septum in order not to induce hippocampal seizures (Ma and Leung, 2014). DBS of the NAC by continuous 130-Hz pulses was also effective to normalize hyperlocomotion and PPI impairment induced by ketamine. DBS may work to inactivate a brain area functionally, or to disrupt the pathological patterns of neural activity (Anderson et al., 2004; McCracken and Grace, 2007).

#### Mechanisms of Generating Ketamine-Induced Gamma Activity

The likely cause of high-amplitude hippocampal gamma after ketamine is an open channel block of NMDA receptors on parvalbumin-positive GABAergic interneurons (Grunze et al., 1996; Widman and McMahon, 2018; Hudson et al., 2020), which increased firing of pyramidal cells by disinhibition. NR1f/f mice with selected ablation of NMDA receptors on parvalbumin-positive interneurons showed a profile of enhanced CA1 gamma waves at 60–100 Hz (Korotkova et al., 2010; Carlen et al., 2012), similar to that induced by ketamine. In addition, the theta and gamma during walking NR1f/f mice were sensitive to atropine (Korotkova et al., 2010), similar to the theta and gamma after PCP (Vanderwolf and Leung, 1983; Leung, 1985).

Neymotin et al. (2011) proposed that ketamine primarily blocks NMDA receptors on oriens-lacunosum-moleculare (OLM) interneurons that inhibit both the parvalbumin-containing basket cells and the distal dendrites of CA3 pyramidal cells. We suggest that the ketamine-induced gamma in CA1 is mainly generated by the recurrent inhibitory feedback loop in CA3 (Figure 1C), but further modulated by OLM interneurons. To sustain a hippocampal gamma, an intact medial septum is necessary to provide septohippocampal GABAergic inhibition of hippocampal interneurons, and muscarinic cholinergic excitation of pyramidal cells. EC may also continue to drive a distal dendritic gamma after PCP/ketamine administration, although a theta-rhythmic driving of the CA1 distal dendrites by EC is attenuated by PCP (Vanderwolf and Leung, 1983; Leung, 1984; Gu et al., 2017). Gamma recorded in the DG in rats, presumably driven by EC, was greatly enhanced by 25 mg/kg i.p. ketamine (Caixeta et al., 2013).

#### Relevance to Schizophrenia

Ketamine gives an acute, drug-induced model of schizophrenia in humans, but the symptoms induced in schizophrenia patients are similar to the prevalent ones during a psychotic state (Lahti et al., 2001). Increase of 40–85 Hz gamma waves in the electroencephalogram was found to accompany the schizophrenia symptoms induced by 0.3 mg/kg i.v. ketamine in healthy humans (Hong et al., 2010). It is difficult to observe or infer the main symptoms of schizophrenia (hallucinations, delusion, and thought disorder) in animals. Instead, tests of sensorimotor gating and sensory gating indicate that animals, like humans, do not filter (gate) their sensory and sensorimotor signals properly such that perception and motor programs may be distorted and disorganized. Large hippocampal gamma is suggested to indicate a faulty consolidation or retrieval process for hippocampus-stored memory, which can lead to recall of distorted memory, with or without trigger or cue. On the motor side, NAC activity may be similarly disrupted by abnormal inputs from the ventral tegmental area, hippocampus, mPFC and posterior cingulate cortex. Disruption of an associative network in hippocampal CA3 is suggested to underlie hallucination in schizophrenia (Behrendt, 2016).

To our knowledge, septal control of schizophrenia symptoms has not been studied in humans. In animals, DBS of the medial septum or NAC effectively reduces schizophrenia-like symptoms induced by ketamine, without inducing seizures or other abnormal behaviors. Whether DBS (or septal inactivation) that controls schizophrenia-like symptoms may induce adverse response such as cognitive impairment remains to be studied.

### Low-Dose General Anesthetics

Ether, the first general anesthetic discovered, and other volatile anesthetics, are known to induce behavioral confusion or delirium during an early stage (stage II) of anesthesia (Guedel, 1951); the delirium ends with sedation induced by the anesthetic. In animals, the period of delirium is accompanied by increased hippocampal gamma waves of 30–50 Hz (Ma et al., 2002; Ma and Leung, 2006), accompanied by ataxic movements. Other than ether, nitrous oxide, halothane, sevoflurane or isoflurane also induces a delirium stage.

Injectable general anesthetic, at a subanesthetic dose, or during anesthesia induction, also induces behavioral hyperactivity in animals. PCP and ketamine were initially designed as injectable general anesthetic, but they induce psychosis-like behaviors at a low dose, as described above. GABA_A_ receptor targeting anesthetic, such as diazepam, pentobarbital and propofol, when administered intraperitoneally, could induce a short period of hyperactivity with ataxic walking, accompanied by increased 20–50 Hz (beta and low gamma) activity in the hippocampus and neocortex.

The behavioral hyperactivity induced by a volatile or injectable anesthetic was suppressed when the medial septum or hippocampus was inactivated by muscimol (Ma et al., 2002). Attenuation of an anesthetic-induced behavioral hyperactivity was also found after inactivation of the NAC, ventral pallidum, ventral tegmental area and supramammillary area (Ma and Leung, 2006), and generally by inactivation of the piriform cortex and EC (Long et al., 2009).

## Conditions That Depress Hippocampal Gamma Activity

### General Anesthetics

Surgical anesthesia induced by a general anesthetic has been observed to decrease high-frequency gamma activity in the hippocampus of animals (Ma et al., 2002; Long et al., 2009; Leung et al., 2014). Sevoflurane anesthesia depressed 30–150 Hz gamma activity in the temporal lobe during loss of consciousness in humans (Uchida et al., 2000). A dose response study in rats indicated that 70–140 Hz gamma activity in the hippocampus, frontal and visual cortices decreased with >0.4% isoflurane (Hudetz et al., 2011). A lower isoflurane dose of 0.25% was found to decrease hippocampal gamma activity of 50–200 Hz recorded at CA1 stratum radiatum during immobility, while higher (0.5 and 1%, respectively) doses were needed to decrease delta (0.8–4 Hz) waves and low-frequency (30–50 Hz) gamma (Kuo and Leung, 2017).

General anesthetics other than isoflurane also decreased hippocampal gamma (62–100 Hz) power when animals were sedated (Ma et al., 2002). The anesthetics included volatile anesthetics halothane and ether, and injected anesthetics including those acting on GABA_A_ and NMDA receptors. At a low, non-sedative dose, pentobarbital at 10 mg/kg i.p. (Kuo and Leung, 2017) or diazepam at 2.5 mg/kg i.p. (Van Lier et al., 2004) decreased high-frequency gamma during immobility or sitting.

At near-LORR doses of ketamine in mice, hippocampal gamma power (30–100 Hz) increased compared to pre-drug baseline, but the coherence between CA1 alvues and frontal cortex decreased (Leung et al., 2021). This was consistent with an increase in 65–100 Hz gamma coherence between electrodes in frontal, parietal and visual cortices as rats recovered from ketamine-induced unconsciousness (Pal et al., 2015). In the case of ketamine or PCP anesthesia, evaluation of functional connectivity using gamma-frequency coherence may be a more useful measure of cortical function than gamma power.

### Local Brain Inactivation Suppresses Hippocampal Gamma Activity and Increases Potency of a General Anesthetic

Local inactivation of the medial septum suppresses spontaneous movements and decreases hippocampal theta and gamma activity. The decrease in hippocampal gamma was statistically different following muscimol, compared to vehicle infusion, at 30–120 min post-infusion (Ma and Leung, 2007; Figure 3E). Inactivation of the medial septum or the hippocampus increased the time durations of LORR and loss of tail-pinch response following an anesthetic dose of halothane, isoflurane, pentobarbital and propofol (Ma et al., 2002).

Inactivation of several wake-active brain areas that project to the medial septum and hippocampus decreases both hippocampal and neocortical gamma activity and increase neocortical delta waves (Ma and Leung, 2006, 2007; Long et al., 2009). The brain areas that suppress hippocampal gamma waves when inactivated include supramammillary area (Ma and Leung, 2007), NAC, ventral tegmental area and ventral pallidum (Ma and Leung, 2006), piriform cortex and EC (Long et al., 2009). After inactivation of each of the above areas, the potency of a subsequently administered general anesthetic was potentiated, as shown by increased durations of LORR and loss of tail-pinch response (Ma and Leung, 2006; Long et al., 2009).

Gamma-butyric hydroxylactone (GBL) decreased locomotor activity and hippocampal gamma activity in rats (Arcaro et al., 2016). GBL induces immobile staring associated with high 2–6 Hz spike-wave discharge, an animal model of absence seizure. Muscimol inactivation of the hippocampus decreased local gamma and increased the decline of gamma power and spike-wave frequency by GBL (Arcaro et al., 2016). The latter results are consistent with an increased potency of the hypnotic effects of GBL following hippocampal inactivation.

Electrolytic lesion of the medial septum reproduced the result of medial septal inactivation. At 10–14 days after an electrolytic lesion of the medial septum, the theta rhythm and a cholinergic marker (acetylcholinestarse stain) in the hippocampus were abolished and hippocampal gamma power was decreased by 2.5-fold (Leung et al., 2013). Septal lesion compared to control rats showed a prolonged duration of LORR and loss of tail-pinch response following halothane, isoflurane or propofol. Lesion compared to control animals showed an increase in anesthetic sensitivity, indicated by a lower ED50 (effective dose for 50% of population) of the LORR to isoflurane and propofol (Leung et al., 2013).

Rats with selective lesion of septal cholinergic neurons by 192 IgG-saporin (lesioned rats) showed an ED50 (LORR) at 0.62 vs. 0.74% in control, non-lesioned rats (Tai et al., 2014). The lower ED50 (LORR) correlated with a larger decrease of 62–100 Hz (gamma 2), but not 30–58 Hz gamma 1) or delta power in the hippocampus (Tai et al., 2014). At the frontal cortex, delta and gamma 2 power change with isoflurane were not significantly different between lesioned and control rats (Tai et al., 2014). Similar results were found in mice depleted of ACh by genetic ablation of the vesicular acetylcholine transporter gene in the basal forebrain, as compared to wildtype mice. Basal forebrain ACh-depleted mice and 192-IgG saporin-lesion rats did not show a significant difference of their hippocampal gamma with respective control animals. However, septal ACh-deficient animals showed a lower ED50 (LORR) in correspondence with a higher decrease of hippocampal gamma 2 power at a pre-LORR isoflurane dose (Leung et al., 2021). These experiments suggest that hippocampal gamma 2 power decrease is correlated with LORR, a surrogate for loss of consciousness in animals.

In contrast to the brain areas reviewed above, muscimol inactivation of the median raphe induced behavioral hyperactivity and did not decrease gamma power in the hippocampus (Ma and Leung, 2006). Inactivation of median raphe did not significantly affect the LORR duration induced by halothane or pentobarbital anesthesia (Ma and Leung, 2006).

## Conclusion: Septohippocampal-NAC System, Consciousness and Psychotic Behaviors

Hippocampal gamma waves are found to correlate physiology and behavior that range from loss of consciousness at the low end and psychotic behaviors at the high end. Gamma waves offer a window into hippocampal neural processing. A low amplitude gamma indicates a lack of hippocampal neural activity and severely limited processing during general anesthesia. Increase in cortical activation and gamma waves in normal animals results in increased performance in sensory perception and motor tasks (Singer, 1999; Sederberg et al., 2007; Sohal et al., 2009; Pinto et al., 2013). However, when hippocampal inhibition is reduced by a seizure or by ketamine administration, high activation is accompanied by abnormally high-amplitude hippocampal gamma waves, which are associated with psychosis-related behaviors.

Increase in hippocampal gamma is not necessary or sufficient for behavioral hyperactivity. In animals under general anesthesia with ketamine, hippocampal gamma was large while the animals were immobilized (Vanderwolf, 2000; Hakami et al., 2009; Leung et al., 2021). In amphetamine models of psychosis, hippocampal gamma was not significantly higher than that during baseline walking (Ma and Leung, 2002; Ehrlichman et al., 2009). Infusion of MK-801 into the NAC or ventral tegmental area induced PPI impairment (Narayan et al., 1996). Infusion of ketamine into the posterior cingulate cortex in rats induced local gamma waves and behavioral hyperactivity, even after muscimol inactivation of the dorsal hippocampus (Ma and Leung, 2018). However, in the hippocampal AD or systemic ketamine model of psychotic behaviors, the correlation of hippocampal gamma with behavioral hyperactivity is consistent with the hypothesis that the hippocampus contributes to the behavioral hyperactivity through the NAC.

A hippocampal AD in animals spreads bilaterally to connecting structures (septum, subiculum, EC, and amygdala), but high-amplitude gamma appears to be restricted to CA1/CA3. Systemic ketamine injection induces high-amplitude gamma in many brain areas, including the hippocampus, mPFC, NAC, cingulate cortex, and different neocortices. However, the experimental result that inactivation of the medial septum (and selective lesion of septal GABAergic neurons) abolishes the increase of hippocampal gamma and psychotic behaviors strongly suggests a critical role of the medial septum on the hippocampus (Swanson and Cowan, 1979). The other two limbic cortices that receive afferents from the medial septum, namely EC and cingulate cortex (Gaykema et al., 1990), may also be involved. Abundant literature has documented medial septal modulation of hippocampal theta and gamma rhythms in normal animals (Stewart and Fox, 1990; Bland and Colom, 1993; Buzsáki, 2002; Csicsvari et al., 2003).

The mechanisms whereby inactivation of the medial septum, or other brain area, attenuate hippocampal gamma waves and psychotic behaviors after ketamine (or seizure) are not completely known. Infusion of 0.5 μL of muscimol (1 μg/ μL) with a fluorescent tag gave an asymmetric spread of ~1 mm (Allen et al., 2008), which may affect non-target neighboring areas. We suggest that septal infusion of a low-dose (0.25 μg) muscimol preferentially works on septal GABAergic neurons, since orexin-saporin lesion of the septal GABAergic neurons also attenuated the ketamine-induced gamma and behaviors (Ma et al., 2012). Thus, inactivation of perifornical n. in the hypothalamus that projects orexinergic afferents to the medial septum is expected to attenuate ketamine-induced gamma and behaviors, as had been shown for inactivation of the supramammillary n. that projects glutamatergic afferents to the medial septum. The participation of septohippocampal glutamatergic neurons in ketamine-induced gamma and behavior is not clear. Septohippocampal glutamatergic afferents are shown to excite CA3 pyramidal cells (Robinson et al., 2016) and OML interneurons (Fuhrmann et al., 2015), possibly resulting in larger gamma in CA3. The detailed mechanisms of septohippocampal modulation of gamma and psychotic behaviors remain to be studied.

In addition to psychotic behaviors, the same medial septum-hippocampus-NAC neural circuit contributes to the maintenance of consciousness, as tested with general anesthesia. However, consciousness is maintained by many brain areas other than the septohippocampal-NAC system (Saper et al., 2010; Leung et al., 2014), and it is not abolished by removal of the septum, hippocampus and NAC. As reviewed above, high-frequency gamma in frontal and other cortices is also suppressed during surgical anesthesia. While the septohippocampal-NAC system contributes to loss of consciousness, its unique function is to mediate hippocampal activation and behavioral arousal when motivation and affect are involved, as in emergence (reanimation) from general anesthesia and psychotic behaviors.

In this review, consciousness is defined by a subject's ability to make behavioral responses, such as response to verbal command in humans, and righting response in animals. This may be considered a basic level of consciousness, which could be experimentally observed in animals and humans. Interconnections between brain areas, notably the neocortex or thalamus have been suggested as the basis of consciousness (Alkire et al., 2008), and specific relation to the septohippocompal system is not known. Altered behaviors after seizures or psychmimetic drugs in animals are considered impairment of consciousness. However, the label of impaired consciousness is less important than knowing that the biological basis of these altered behaviors is in the septohippocampal-NAC system.

Inactivation of local brain areas has provided the main evidence of the function of the medial-septum-hippocampus-NAC system. Inactivation of a single brain area within the septohippocampal-NAC system can have various effects: (i) it decreases hippocampal gamma power and spontaneous movements of the animals; (ii) it potentiates the effects of the general anesthetic and delays the recovery of righting reflex from general anesthesia; (iii) it normalizes hippocampal gamma and hyperactive behaviors, and reduces schizophrenia-like symptoms like PPI and auditory gating in animals given ketamine injection or an hippocampal seizure. More detailed connections and functions of the system will be further revealed by optogenetic and chemogenetic techniques.

## Author Contributions

LL wrote most of review. JM and LL co-authored the experimental papers on which the review was based. All authors contributed to the article and approved the submitted version.

## Funding

Research in this review was supported by the Canadian Institutes of Health Research (MOP 15685) and Natural Science and Engineering Research Council of Canada (1037-2008) to LL.

## Conflict of Interest

The authors declare that the research was conducted in the absence of any commercial or financial relationships that could be construed as a potential conflict of interest.

## Publisher's Note

All claims expressed in this article are solely those of the authors and do not necessarily represent those of their affiliated organizations, or those of the publisher, the editors and the reviewers. Any product that may be evaluated in this article, or claim that may be made by its manufacturer, is not guaranteed or endorsed by the publisher.
